# The Impact of the Ovine Annular Lesion Model on IVD Pathobiology and Utility of the Ovine Spinal Model in Patho‐Anatomical Studies: A Historical Perspective

**DOI:** 10.1002/jsp2.70128

**Published:** 2025-11-04

**Authors:** O. Osti, C. B. Little, J. Melrose

**Affiliations:** ^1^ The International Spine Centre Norwood South Australia Australia; ^2^ Calvary North Adelaide Hospital North Adelaide South Australia Australia; ^3^ The University of Adelaide Adelaide South Australia Australia; ^4^ Raymond Purves Bone and Joint Research Laboratory Kolling Institute St. Leonards New South Wales Australia; ^5^ School of Medical Sciences, Faculty of Medicine and Health The University of Sydney at Royal North Shore Hospital St. Leonards New South Wales Australia; ^6^ Graduate School of Biomedical Engineering University of New South Wales Sydney New South Wales Australia

**Keywords:** intervertebral disc degeneration model, IVD histopathology, mesenchymal stromal cells, recovery of IVD composition and function

## Abstract

This review documents the impact of the Osti ovine annular lesion model of intervertebral disc (IVD) degeneration on the elucidation of degenerative changes in the ovine IVD also seen in human IVD degeneration (IVDD). The degenerative pathology knowledge base generated by the ovine model over the last 35 years has provided invaluable insights into degenerative events occurring in the human IVD. Changes in para‐discal structures as a consequence of IVDD, in vertebral bone adjacent to the lesion site, cartilaginous endplates and spinal motion segment facet joints, and in the longitudinal ligaments and paraspinal muscles also affect spinal stability and flexibility. All of these spinal structures are richly innervated, and disturbance of their normal architecture due to IVDD also contributes to the generation of low back pain. Like human IVDD, when the ovine IVD is mechanically destabilized by the introduction of a controlled annular lesion, release of proteases is elevated, degradation of structural ECM components occurs, leading to IVDD and biomechanical impairment. When space‐filling aggrecan is degraded in the IVD, a drop in internal hydrostatic pressure occurs, and the IVD becomes susceptible to an ingrowth of blood vessels and mechanosensitive nociceptors resulting in enhanced generation of low back pain by the biomechanically incompetent IVD. The ovine model is thus very useful to evaluate compounds or procedures that slow IVDD and, in some cases, promote regenerative processes. The large size of the ovine IVD allows the use of inter‐disciplinary longitudinal approaches in the analysis of progressive changes in the degenerate IVD of relevance to the human IVD.

**Clinical Significance:**

The ovine annular lesion model of IVDD displays similar pathological features to those displayed by the degenerate human IVD, making it an appropriate model for the evaluation of IVD reparative procedures. Furthermore, the resident ovine IVD cell populations are similar to those seen in the human IVD, with a disappearance of notochordal cells occurring in adolescence. This is not the case in many other popular animal (murine, rat, porcine, lapine, and non‐chondrodystrophic canine) models of IVDD. The persistence of notochordal cells into adulthood in these breeds questions how translatable findings generated in these models are to the human IVD. The ovine model is thus relevant to the development of strategies exploring novel strategies in IVD repair and the recovery of normal IVD structure and function. Mesenchymal stem cells have impressive IVD repair and recovery of structure and function properties, showing promise in the treatment of the degenerate human IVD.

## Graphical abstract

The ovine annular rim‐lesion model of intervertebral disc degeneration of Osti and colleagues (1990) has evolved into a sophisticated mechanical destabilization large animal model of intervertebral disc degeneration over its 35 year history offering diverse musculoskeletal multi‐disciplinary investigations into the multifunctional complexity of this condition relevant to the human condition.

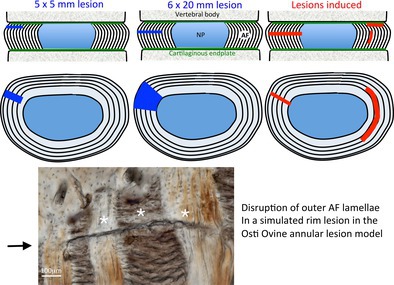

**Abbreviations**
ADAMTS: a disintegrin and metalloproteinase with thrombospondin motifs
AF: annulus fibrosus
Akt1: RAC‐alpha serine/threonine‐protein kinase
BBB: blood brain barrier
BMP: bone matrix protein
CEP: cartilaginous endplate
CS: chondroitin sulfate
ECM: extracellular matrix
FGF: fibroblast growth factor
GAG: glycosaminoglycan
HA: hyaluronan
IGF‐1: insulin‐like growth factor‐1
IL: interleukin
ISSLS: International Society for the Study of the Lumbar Spine
IVDD: intervertebral disc degeneration
JAK2: Janus kinase 2
LBP: low back pain
LOX: lysyl oxidase
LRP‐1: low density lipoprotein receptor‐related protein 1
MAPK: mitogen‐activated protein kinase
MDM2: MOUSE double minute 2 homolog, E3 ubiquitin‐protein ligase
MMPs: matrix metalloproteases
MRI: magnetic resonance imaging
MSCs: mesenchymal stem cells
mTOR: mammalian target of rapamycin
NIH: National Institute of Health
NOC: non‐operated control
NP: nucleus pulposus
p16: a tumor suppressor protein that is a cyclin‐dependent kinase inhibitor
p53: tumor protein P53, tumor antigen p53, transformation‐related protein 53
PCG‐1α: peroxisome proliferator‐activated receptor‐gamma coactivator
PCM: pericellular matrix
PG: proteoglycan
PI3K: phosphatidylinositol‐3 kinase
pSTAT3: phosphorylated signal transducer and activator of transcription 3
RG‐7112: small‐molecule MDM2 inhibitor
rhOP‐1: recombinant human osteogenic protein‐1
ROS: reactive oxygen species
SDC4: syndecan‐4
ShhCreERT2: tamoxifen‐induced sonic hedgehog Cre recombinase
SMA: smooth muscle cell actin
TGF: transforming growth factor
TNF: tumor necrosis factor

## 1 Introduction

### 1.1 Aims of the Study

The aim of this study was to document the Osti annular lesion model of intervertebral disc degeneration (IVDD) since its development in 1990 [1], and to show how it has contributed to our understanding of the pathobiology of IVDD, providing information translatable to the human IVD. This study also demonstrated the utility of the ovine spine as a model for patho‐anatomical spinal studies on musculoskeletal spinal changes that are also affected by IVDD.

### 1.2 The Need for an Animal Model of IVD Degeneration and Low Back Pain

There is a clear need for a reliable IVDD animal model that generates findings translatable to the human IVD. IVDD is a leading cause of low back pain (LBP) in humans and is the single leading cause of musculoskeletal disability worldwide [2]. In 2020, LBP affected 619 million people globally, and the number of LBP cases is estimated to increase to 843 million cases by 2050, driven largely by population expansion and aging [3]. The prevalence of LBP increases with age up to 80 years. The highest incidence of LBP is between the ages of 50 and 55 years and is more prevalent in women than men [4]. Individuals of any age, however, can develop LBP, including adolescents and children. A 10‐year global study of 291 major human diseases established LBP as the number one disabling musculoskeletal condition with major socioeconomic impact [2, 5, 6]. Approximately 80% of the general population will experience LBP of sufficient severity to require intervention by a physician [7]. LBP results in a loss of workdays and is of major economic impact not only on affected individuals but also is a drain on national healthcare resources [2]. In order to study early and progressive stages of IVDD and develop new, effective, and targeted therapies for both IVDD and LBP, a reliable animal model that reproduces the human condition is a global research imperative. This review shows that the ovine annular lesion of IVDD^1^ represents such a model.

## 2 The Normal Human Intervertebral Disc Structure and Function

The IVD is a complex composite load‐bearing structure interposed between the bony vertebral bodies that also provides spinal flexibility [8, 9].

### 2.1 The Nucleus Pulposus and Roles of Associated Tissues in the Composite IVD

The central aggrecan‐rich region of the IVD, the nucleus pulposus (NP), provides weight‐bearing properties to the IVD. Collagenous lamellae of the annulus fibrosus (AF) that surround the NP provide tensile properties to the IVD, resisting shear forces during torsional spinal loading. Bundles of fibrillar type I collagen in the AF insert through the hyaline cartilaginous endplates (CEPs) into the vertebral bodies, firmly anchoring the IVD [10, 11]. Capillary networks from the vertebral bodies penetrating the CEPs have roles in the nutrition of resident disc cell populations and the removal of metabolic waste products. The AF lamellae contain translamellar cross‐bridges which provide cohesive properties [12]. Cells in the AF have elongated interconnected fibroblastic morphologies, while the CEP and NP contain single or clustered chondrocyte‐like cells. NP cells are surrounded by an abundant extracellular matrix (ECM) rich in the chondroitin sulfate proteoglycan aggrecan, which is entrapped within a random type II collagen network [13]. Individual NP cells are surrounded by a pericellular matrix (PCM) composed of lattice‐forming type VI collagen and the heparan sulfate proteoglycan perlecan. The PCM cushions the disc cell, providing protection from excessive mechanical loading, and also provides cell‐ECM communication and osmo‐regulatory mechanotransductive properties, which allow the disc cells to regulate tissue homeostasis and optimize tissue function in response to loading [14, 15]. Aggrecan interacts with hyaluronan (HA) via its N‐terminal G1 domain to form massive space‐filling aggrecan‐HA macroaggregates. The carboxyl terminal G3 domain of aggrecan interacts with structural glycoproteins and collagen networks to form an extended mechanoresponsive network extending into the interstitial matrix [16]. These aggrecan‐HA‐collagen networks allow disc cells to perceive and respond to perturbations in their extracellular microenvironment to regulate tissue homeostasis. Perlecan also co‐localizes with elastin fibers [15] which localize parallel to collagen fibers [17, 18] providing viscoelastic properties to the AF [19] and recoil properties to the collagen fibers of the AF [18]. The facet joints of the spine stabilize spinal motion segments, and along with the anterior and posterior ligaments and paraspinal muscle networks, stabilize the spine and provide spinal flexibility. In the healthy spine, facet joints constrain the total range of spinal motion and transfer loads in the axially loaded IVD [20, 21, 22].

### 2.2 The CEP is an Important Functional Component of the Composite Disc Structure

**FIGURE 1:**
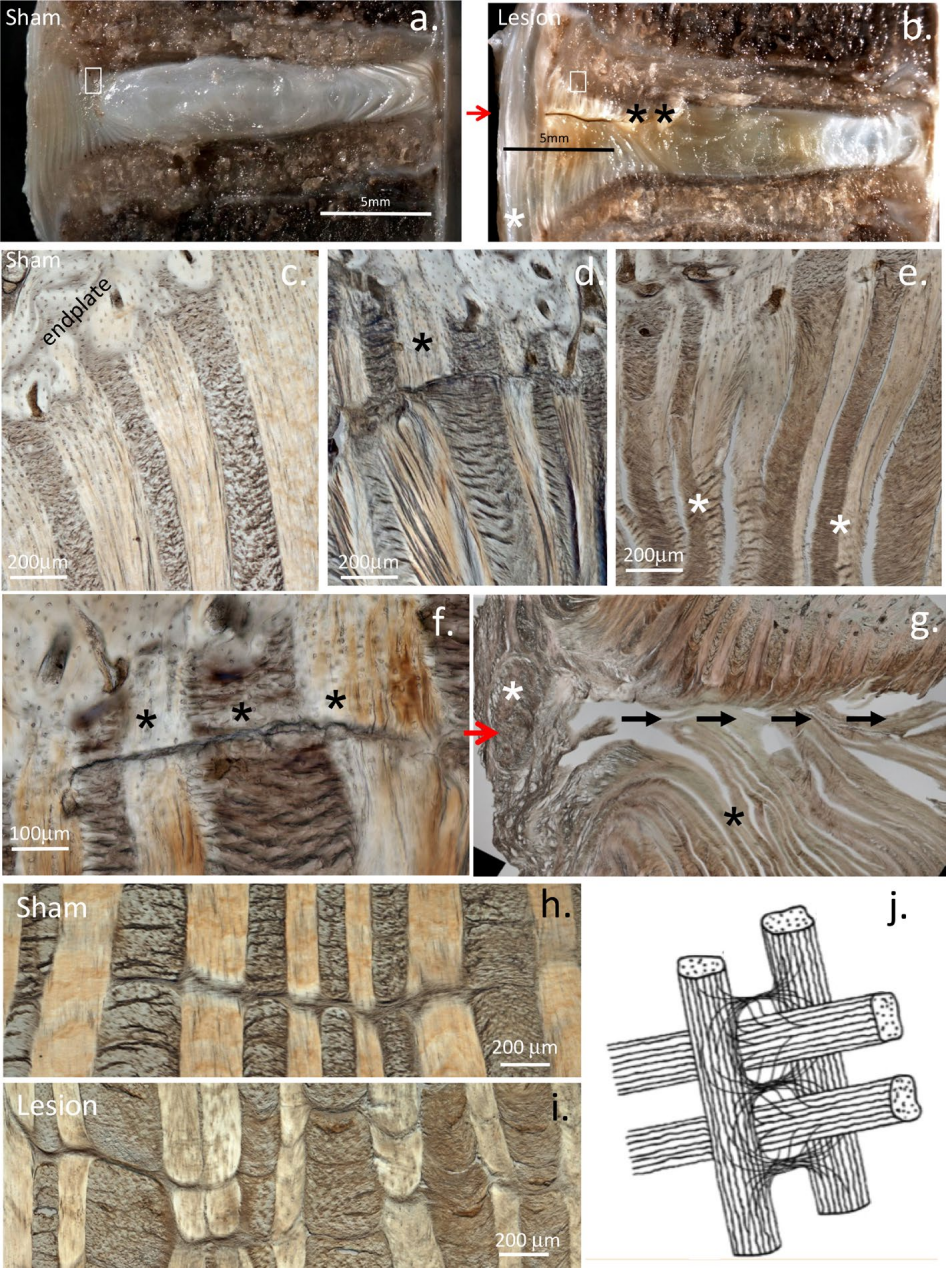
Lesion and sham‐operated IVDs of Merino wethers were subjected to en face oblique and vertical sectioning. The macrostructural effect of the destabilization procedure was examined in the vertical sections (a, b). The oblique sectioned IVD group showed the annular microstructure in its fully hydrated state using a differential interference contrast microscope (c–i). Normal CEP attachment region (c) showing the alternating in‐plane and cross‐sectioned annular lamellae in the attachment zone containing Sharpey's fibers. An annular rim‐lesion with disruption in the endplate attachment region (*) (d), de‐lamination of annular lamellae (*) (e), and a defect spanning across adjacent lamellae (*) (f). Macroscopic disruption of annular lamellae in lesion affected IVD (g). Translamellar cross‐bridge formations in sham IVDs spanning 7 annular layers (h). Finer but less continuous translamellar cross‐bridges are evident in lesion affected IVDs (i). Presentation of an artistic impression of the interrelationship between annular lamellae interconnected by translamellar cross bridges (j). Figure reproduced from [32] with permission.

**FIGURE 2:**
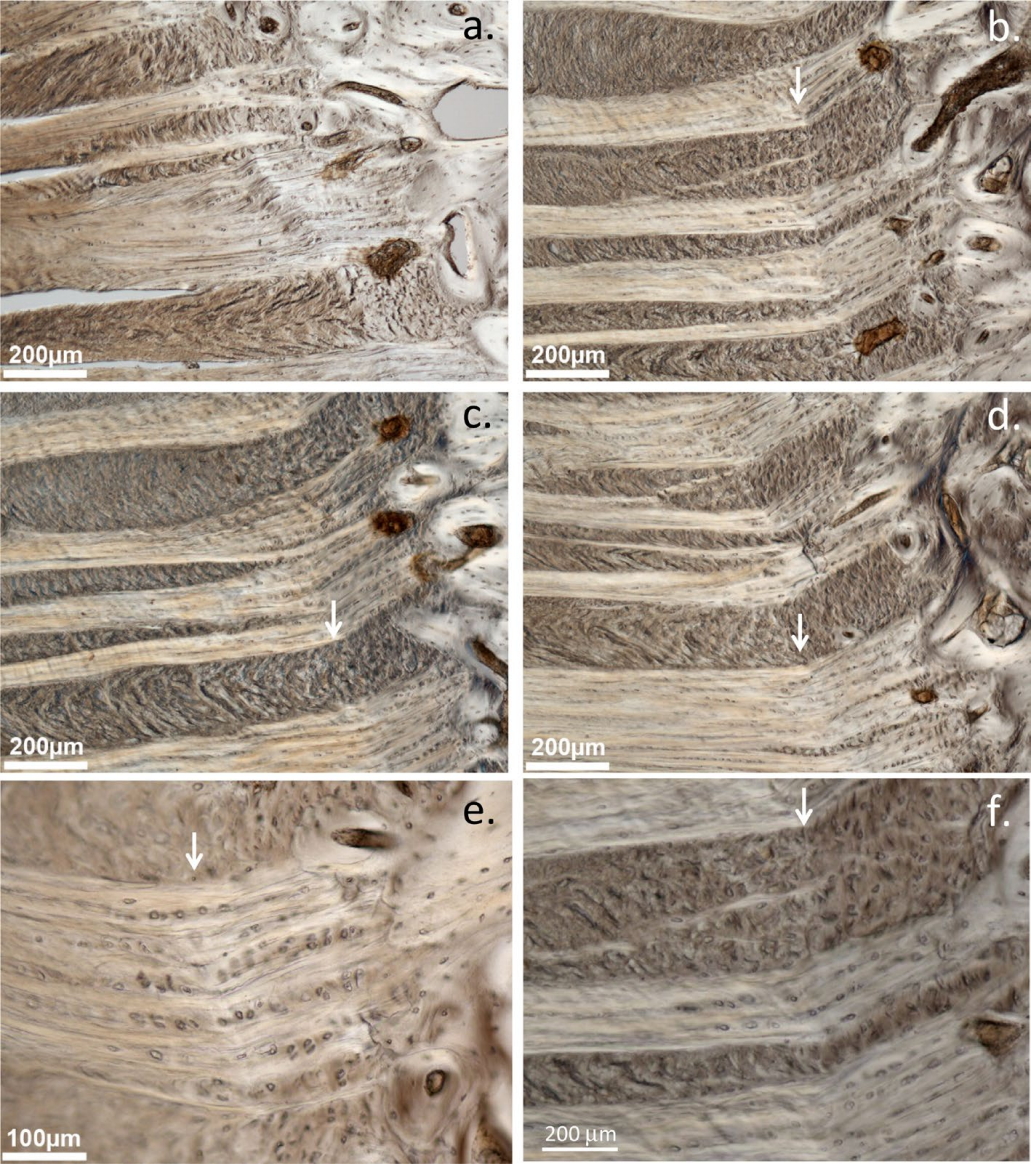
Disturbance of the annular attachment region to the CEP in 6 × 20 mm lesion‐affected ovine IVDs. Oblique‐sectioned IVDs. A kink in the annular attachment adjacent zone to the CEP is labeled with white arrows. Figure reproduced from [32].

**FIGURE 3:**
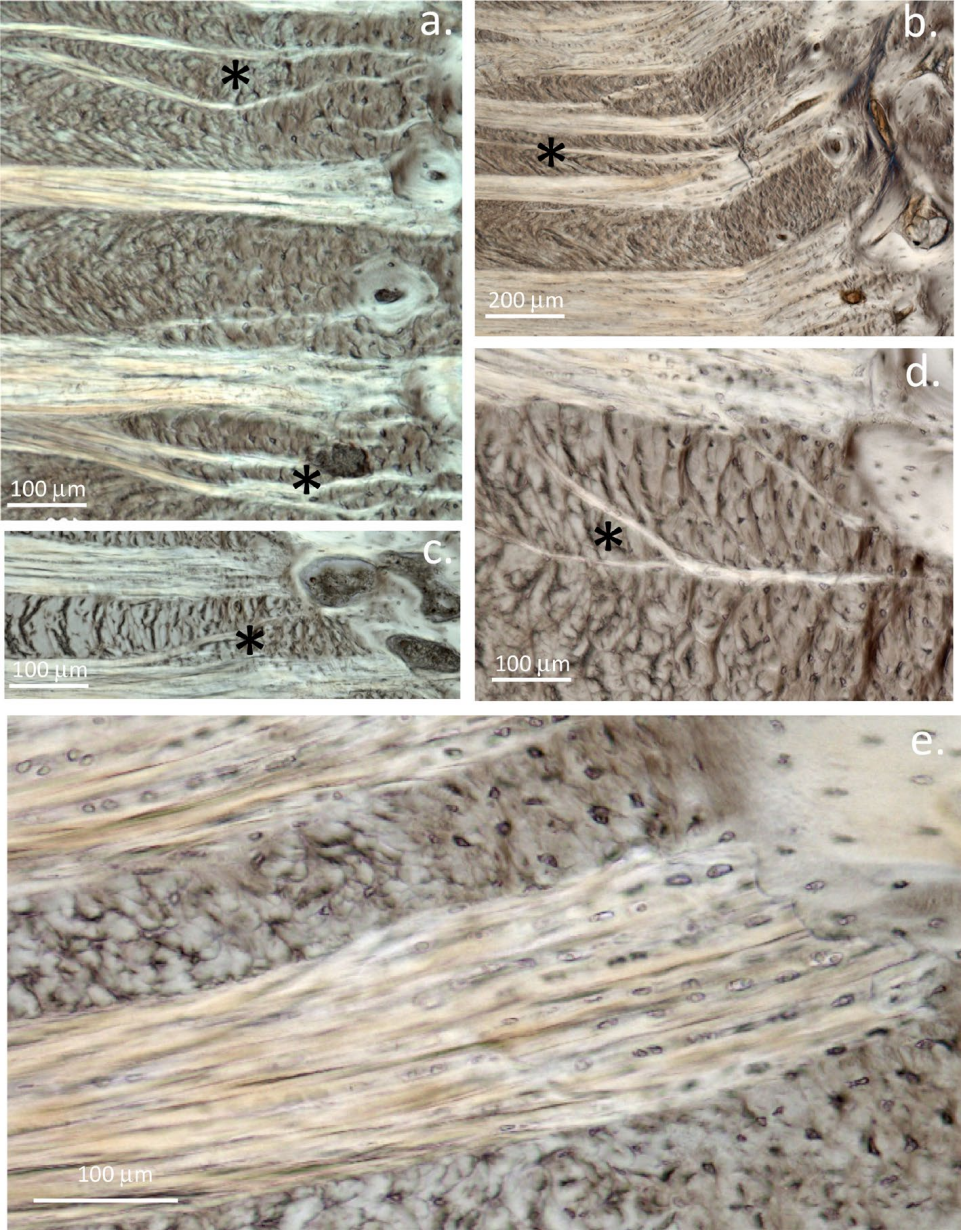
Abnormal branching of collagenous fiber bundles in the annular attachment region to the endplate (*) in ovine IVDs affected by outer annular lesions (a–d). A higher magnification of a normal attachment region is also shown (e). Annular layers are shown as cross‐sections and in‐plane formations. Figure reproduced from [32].

The CEPs interface with the vertebral bodies and distribute disc mechanical loads to the vertebral bodies of the spine during axial spinal loading. Collagenous fibers in the IVD anchor the disc to the vertebral bodies through the CEP, providing mechanical stabilization [23]. Histological examination of the CEP using differential interference contrast microscopy demonstrates intricate attachments anchoring the IVD to vertebral bone [24] (Figures 1, 2, 3). These figures illustrate changes seen in the ovine IVDD model but are representative of degenerative changes reported in the degenerate human IVD [25, 26, 27]. Ultrashort echo time MR imaging has also been used to image this attachment zone in human IVDs [10]. Loading induced changes to native collagenous attachments and microstructural damage in the CEP have been demonstrated in IVDD [28], emphasizing the IVD, CEP, vertebral bone marrow, and vertebral bone as a functional unit during normal spinal loading [29, 30, 31].

### 2.3 The Contribution of the Facet Joints to Spinal Stability

The zygapophyseal or facet joint is a diarthrodial joint enclosed by a ligamentous capsule. The facet joints, together with the IVDs and spinal ligaments, couple adjacent vertebrae in all spine regions, providing control of load transfer in the spinal column and have a collective role in the provision of spinal stabilization [33, 34, 35]. The facet joints carry between 20% and 30% of the healthy spinal motion segment's axial load [36, 37] and also contribute up to 48% of the motion segment's torsional stiffness during normal bodily movement [36]. When the lumbar IVD undergoes degenerative change, up to 70% of the compressive load can be transmitted across the facet joint, which may lead to compressive overload of spinal structures [38].

### 2.4 Molecular Changes That Occur When the Human IVD Undergoes Degeneration

**FIGURE 4:**
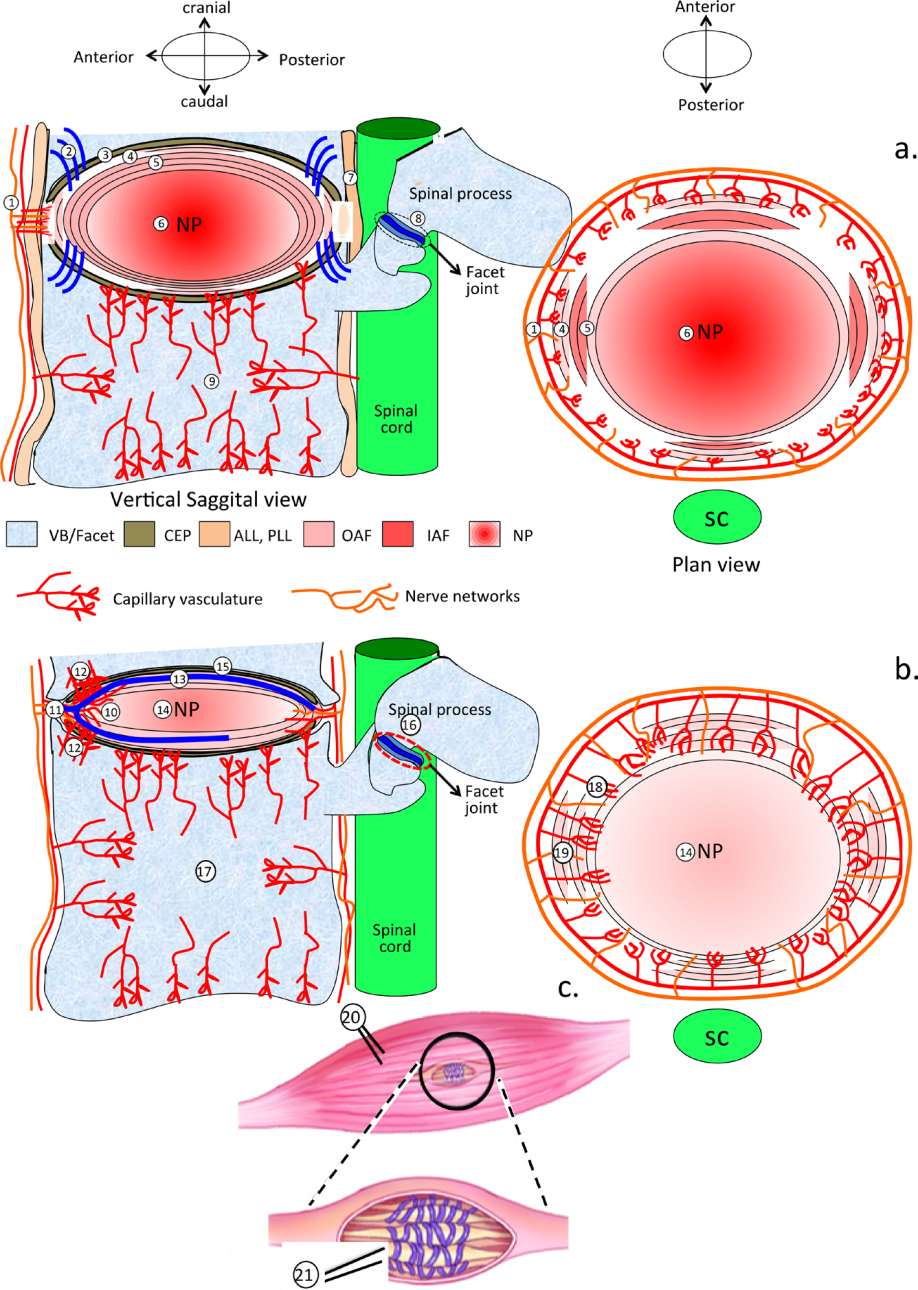
A schematic summation of the structural organization of the normal IVD and paraspinal structures (a) and structural reorganization in spinal tissues induced by a controlled annular lesion in the ovine model (b). (1) The sparse blood vessel and nerve supply to the outer annular lamella in a normal IVD. (2) Sharpey's fibers of that anchor the outer AF to the end plate and vertebral body. (3) Cartilaginous endplate. (4) Outer annular lamellae. (5) Inner annular lamellae. (6) Nucleus pulposus. (7) Posterior longitudinal ligament. (8) Facet joint of spinal processes. (9) Capillary network of the vertebral bodies. (10) Ingrowth of blood vessels into the degenerate IVD. (11) Nerve ingrowth into the degenerate IVD. (12) Elevated vascularization of the vertebral bone adjacent to annular rim lesions. (13) Annular lesion propagating through outer AF towards the contralateral. (14) The proteoglycan depleted degenerate NP in a degenerate IVD of reduced disc height. (15) Reduction in the capillary network supplying the endplate in the degenerate IVD. (16) Osteoarthritic facet joint of spinal process in the degenerate IVD. (17) Reduction of capillary network in the vertebral body of degenerate IVD. (18) Ingrowth of capillary blood vessel network from outer AF in the degenerate IVD. (19) Ingrowth of nerve fibers from outer AF in the proteoglycan depleted degenerate IVD. (20) An example of a spinal muscle (multifidis). (21) Structural alteration in spinal muscle spindles with IVDD. ALL, anterior longitudinal ligament; CEP, cartilaginous endplate; IAF, inner annulus fibrosus; NP, nucleus pulposus; OAF, outer annulus fibrosus; PLL, posterior longitudinal ligament; SC, spinal cord.

Aging and trauma in the IVD result in increased production of inflammatory mediators such as tumor necrosis factor (TNFα, interleukin‐1 (IL‐1β and IL‐6) [39]). These cytokines stimulate the production and secretion of matrix metalloproteases (MMPs) and A Disintegrin and Metalloproteinase with Thrombospondin motifs (ADAMTS) 4 and ADAMTS5 [40] which can degrade IVD ECM components [41]. Degradation of aggrecan, the major disc proteoglycan, results in progressive dehydration of the IVD and a significant reduction in its viscoelastic hydrodynamic weight‐bearing properties [42]. Degenerate IVDs with reduced aggrecan content are susceptible to an ingrowth of blood vessels, nociceptors, and mechanoreceptors, which are normally confined to the outermost lamella in normal IVDs [43, 44, 45] (Figure 4). This results in the biomechanically incompetent IVD containing mechanosensitive nociceptors acting as a significant generator of LBP. Furthermore, IVDD causes alterations in the normal architecture of paradiscal myotendinous tissue, vertebral body, and facet joints. These tissues also contain nociceptive mechanoreceptors and contribute to the generation of LBP [46].

## 3 The Ovine Annular Lesion Model of IVDD

### 3.1 The Importance of Merino Genetics in the Development of a Model of IVDD

Sheep domestication from its wild ancestor in central Asia, the mouflon ( *Ovis orientalis* ), began more than 11,000 years BC [47]. Ciani et al. and the sheep genomics consortium (2015) have examined the genetic diversity, structure, and traits of Merino and Merino‐derived breeds [48]. The ovine genome has been published by The International Sheep Genomics Sequencing Consortium [49]. The well‐defined genetics of the Australian Merino is a major strength in its use as an animal model for the examination of musculoskeletal disorders and minimizes biological variability in the data generated.

### 3.2 A Brief History of the Ovine Model

The ovine annular lesion model of IVDD had its origins in observations made on a series of 135 human cadaveric spines where annular rim lesions were observed in adolescents who had no overt degenerative changes elsewhere in the IVD [50, 51]. This led to the hypothesis that damage to the AF attachments to the vertebral body (rim lesions) could be an initiating factor in the development of IVDD. This went against the existing dogma at this time, which considered that degenerative changes initiated in the NP led to degenerative changes in the AF. Many animal models of IVDD exist [52, 53, 54, 55] however, of the large animal models of IVDD so far developed, the ovine model stands out as being significant in terms of similarity in structure to human IVDs, the spectrum of human IVD degenerative features it reproduces, and the cytomorphology of its resident cell populations [1, 56, 57, 58, 59].

### 3.3 Development of the Ovine Annular Lesion Model of IVDD

The sheep has proven to be an extremely useful large animal model of IVDD [52, 53, 56]. Spatiotemporal changes in discal and paradiscal components in the NP [56], CEPs [60], facet joints [61], and vertebral bone occur adjacent to and distant from the annular lesion site used to induce IVD degeneration [62].

The original Osti model utilized a 5 × 5 mm controlled outer anterolateral surgical lesion to induce IVDD over a 24‐month period. This was modified in 2012 by Melrose and colleagues to a 6 × 20 mm annular lesion which induced IVDD over a shorter time scale (3–6 months) [57]. Severe disruption in normal annular architecture and mechanical destabilization of the IVD was evident, but this did not produce prolapse of the NP (Figures 1, 2, 3). Altered gene expression profiles also occurred, with the expression of MMP‐1, 13, ADAMTS4, and ADAMTS5 elevated. MMPs are active players in IVDD and degrade proteoglycans responsible for the weight‐bearing and viscoelastic properties of the IVD [40, 63, 64]. MMP dysregulation is a crucial factor in the development of IVDD; a more complete understanding of MMP IVD degradative mechanisms is essential in the development of any prospective therapies that aim to successfully target IVDD [41]. When the degenerate IVD becomes depleted of aggrecan, it becomes susceptible to the ingrowth of blood vessels and nerves [65]. Focal expression of FGF‐2, TGF‐β1, and α‐smooth muscle cell actin [66] by cell populations associated with annular remodeling and attempted repair of the lesion site has also been observed, but this repair process is incomplete.

### 3.4 Degenerative Effects Mediated Through CEP‐IVD‐Facet Joint Cross‐Talk

Structural investigation of the annulus‐endplate anchorage system and its mechanisms of failure has been investigated in sheep [32, 67]. IVD and vertebral endplate subchondral bone changes are associated with Modic‐1 changes in the lumbar spine [68]. Some of these anchoring annular collagenous fibers in the CEP are termed Sharpey's fibers; these can become calcified anchorage points to the vertebral body.

A study in rabbits [69] showed that the CEP, IVD, and facet joints communicate with one another and that when the CEP of the IVD is injured, abnormal stresses can be transferred through the IVD to the facet joints and paradiscal structures with deleterious effects on spinal flexibility and IVD functional properties, including pain generation. This has led to proposals that manipulation of compression, flexion, and facet‐constrained shear forces may represent a more physiologically relevant method for induction of IVDD involving the CEP, IVD, and facet joints [70, 71]. However, early studies using the Osti annular lesion model had already demonstrated that annular rim lesions could generate changes in the CEP and facet joints as part of the IVD degenerative process [31, 61, 62].

### 3.5 IVDD Effects Spinal Flexibility and Paraspinal Myotendinous Pathophysiology

The multifidus is a short, strong muscle that stabilizes vertebral movements relative to one another during normal spinal flexion, relaxation, and rotation, which occurs with normal bodily movements. Deep spinal muscles (*transversospinales*), including the semispinalis, multifidis, and rotatores, stabilize the spine, assisted by the longitudinal anterior and posterior ligaments [72, 73]. The multifidus has an important role to play in spinal stability [74], with degeneration of these muscles causing long‐term disability and LBP [75]. All of these components and the degenerate IVD are highly innervated and can generate LBP [46, 76]. When one of these functional groups is dysfunctional, there are compensatory changes in other components, such as the deep spinal muscles [58, 59, 77, 78, 79]. A number of studies have been conducted on the multifidis muscle of sheep, where IVDD was induced by a controlled annular lesion [77, 78, 79, 80, 81]. The proportion of slow multifidis muscle fibers has been shown to be significantly less in annular lesion sheep, especially in the deep medial muscle region [77]. Muscle fiber changes that occur after the induction of an IVD lesion are accompanied by a parallel increase in TNF‐α and IL‐1β expression. Changes in proinflammatory cytokine levels may be a novel mechanism whereby behavioral and structural changes in the multifidus occur. Increased adipose and connective tissue (fibrotic proliferation) and a transition from slow‐to‐fast muscle fiber transition in the multifidis of sheep is induced by IVDD [78]. Increases in effector gene expression provide a putative mechanism for multifidus structural remodeling. A rat model of IVDD has been developed by surgical resection of the multifidus; this induced spinal instability [82] and this also demonstrated the interdependence of spinal muscles and IVDs in the provision of spinal stability [83]. Injury of the anterior longitudinal ligament can also lead to IVDD [84].

Table 1 documents the many degenerative features of the ovine model of IVDD induced by rim lesions, which are also characteristic of degeneration of the human IVD, demonstrating the appropriateness of the ovine model for translation to the human condition.

**TABLE 1:** Studies conducted between 1990 and 2025 using the ovine annular lesion model showing how it reproduces features found in human IVDD and impacts on paradiscal tissues and spinal flexibility.

Date	Study features	References
1990	1990 Volvo Award in experimental studies. Annulus tears and intervertebral disc degeneration. An experimental study using an animal model.	[1]
1992	Progressive longitudinal changes in the GAG composition of IVDs in outer annular lesion affected IVDs in the lesion affected zone but not in the contralateral AF.	[56]
1996	Local structural remodeling of vertebral bone and Modic changes next to the outer annular lesion site and an influx of blood vessels into the CEP next to the lesion site.	[62]
1997	Up‐regulation in MMP expression, disruption in normal levels of IVD proteoglycan and collagens and catabolism of ECM components.	[85]
1999	OA like degenerative changes in the facet joint capsule.	[61]
2002	Ingrowth of blood vessels and nociceptive mechanoreceptors into the degenerate ovine IVD depleted of its space‐filling aggrecan content induced by controlled outer annular lesions.	[65]
2002	Focal expression of FGF‐2, TGF‐β1, and α‐smooth muscle cell actin by cells associated with annular remodeling and attempted repair of the lesion site.	[66]
2007	Fragmentation of biglycan and fibromodulin in ovine outer annular lesion site.	[86]
2008	Potential of AF lesions to undergo repair.	[87]
2008, 2009	Microstructural analysis of normal and internal mechanically disrupted trans‐lamellar bridging networks in the ovine model.	[88, 89]
2010	A detailed microscopic examination of alterations in normal annular structure induced by mechanical destabilization in an ovine model of disc degeneration.	[32]
2012	Elevation in MMP‐1, 13, ADAMTS4, and ADAMTS5 expression in ovine IVDs that received controlled 6 × 20 mm annular lesions.	[57]
2012	Mechanical destabilization by a 6 × 20 mm annular lesion induces IVDD.	[57]
2012 2015 2017	Microscopical analysis of CEP structural attachments to vertebral bone in the ovine IVD, mechanisms of failure, and structural stabilization in skeletal maturity.	[67, 90, 91]
2014	Proinflammatory cytokine gene expression induced by IVDD in the multifidus results in muscle fiber changes and effects on spinal flexibility.	[77]
2015	Multifidus muscle changes induced by IVDD result in structural remodeling of muscle, adipose and connective tissue, but do not result in muscle atrophy.	[78]
2016	Mesenchymal stem cell treatment of IVDD prevents fatty infiltration and fibrosis of the multifidus muscle, but not cytokine and muscle fiber changes.	[79]
2017	A more realistic disc herniation model incorporating compression, flexion and facet‐constrained shear: a mechanical and microstructural analysis. Part I: Low rate loading.	[71]
2017	A more realistic disc herniation model incorporating compression, flexion and facet‐constrained shear: a mechanical and microstructural analysis. Part II: high rate or “surprise” loading.	[70]
2017	A Histopathological scheme for the quantitative scoring of intervertebral disc degeneration and the therapeutic utility of adult mesenchymal stem cells for intervertebral disc regeneration.	[58]
2018	Three‐dimensional microstructural reconstruction of the ovine IVD using ultra high field MRI.	[69]
2018	Efficacy of administered mesenchymal stem cells in the initiation and co‐ordination of repair processes by resident disc cells in an ovine ( *Ovis aries* ) large destabilizing lesion model of experimental disc degeneration.	[59]
2018	IVDD induces macrophage polarization contributing to local myo‐tendinous inflammation and structural change in the multifidus muscle.	[80]
2022	Muscle spindles of the multifidus muscle undergo structural change after IVDD.	[81]
2024	Targeted multifidus muscle activation reduces fibrosis of multifidus muscle following intervertebral disc injury.	[92]
2025	ISSLS Prize in Basic Science 2025: Structural changes of muscle spindles in the multifidus muscle after intervertebral disk injury are resolved by targeted activation of the muscle.	[93]

## 4 Similarities in Sheep and Human Spinal Components

Sheep and human spines show close similarities in terms of gross anatomy. Sheep and human vertebrae are most similar in the thoracic and lumbar regions, but show substantial differences in certain dimensions [94, 95]. Spinal level morphological variations are well matched in the two species; however, substantial differences in the angulations of the facet joints are evident, as well as in the shape and length of the spinal processes. The sheep spine is horizontally loaded, while the human spine is vertically loaded. Even so, the sheep is the only animal species where Schmorls nodes have been observed other than humans; thus, quadrupedic spinal loading does not alter the locations where these occur, showing similarities in spinal loading [96]. The human spinal canal is wider and deeper in the anteroposterior plane compared to the sheep [94]. In terms of their biomechanical parameters, the sheep and human spines are similar in terms of range of motion, neutral zone, and stiffness parameters in the complete cervical, thoracic, and lumbar spines in flexion and extension, axial left/right rotation, and right/left lateral bending [97].

### 4.1 Imaging and Flexibility Measurements of the IVD

Quantitative anatomic, radiographic, computerized tomographic, and biomechanical data of sheep and human cervical spines have been compared [98]. Range of motion differed significantly between the two species except in flexion‐extension, axial rotation, and lateral bending. Bone mineral density in the human cervical spine has been shown to be fourfold higher than in the sheep cervical spine [98]. Despite minor differences between human and sheep cervical spines, the good comparability with the human spine makes the sheep cervical spine a useful model for cervical spine research [99]. A parametric finite element model of the L4–5 spinal motion segment has also been developed to test the geometry and material properties of the *human* and *ovine* spine under pure and combined loads, and whether disc degeneration can be depicted [100, 101]. This model highlighted potential differences in the mechanical response to disc degeneration between *human* and *ovine* IVDs and emphasized the need for further development of the model to resolve this issue [99]. Overall, however, the above findings indicate the sheep spine is a useful model of the human spine. The sheep IVD shows close similarity to the human IVD in terms of structural organization in its annular components and the collagenous organization and proteoglycans present in the NP [102]. The ovine CEP also shows strong parallels in structure and function to its human counterpart, making the sheep a useful model of the human IVD. Moreover, the cell populations in the ovine IVD are also similar to those found in the human IVD [103]. This is not always the case in all large animal IVDs, particularly in the persistence of notochordal cell populations and the rate of maturational changes that occur in IVD tissues or their responses to mechanical destabilization.

## 5 The Utility of the Ovine Spine Model for Patho‐Anatomical Studies

Table 2, part A illustrates the many studies that have utilized the ovine model of experimental disc degeneration, and other segments in this table illustrate the ovine spine in a diverse range of patho‐anatomical examinations. These studies clearly show how the ovine lesion model has significantly aided in the elucidation of multiple facets of disc degeneration induced by a destabilizing defect in the annular attachments to the vertebral bodies in the CEP and emphasizes the interdependence of multiple tissue components in IVD degenerative processes. Table 2 shows how endplate vascularity is adversely affected by rim lesions [31] which induce longitudinal compositional changes in affected IVDs [56] and vertebral bone undergoes remodeling in the vicinity of rim lesions [62]. Aggrecan is selectively degraded in the ovine model [85] and OA changes in facet joints also occur [61]; both of these contribute to IVDD. Concentric, radial, and circumferential tears also occur [104] as a consequence of rim lesions in annular tissues, further reducing the mechanical properties of degenerate IVDs. An influx of blood vessels [65], noci‐ and mechanoreceptors occurs in degenerate IVDs [65] depleted of aggrecan, and cell populations expressing FGF‐2, TGF‐β, and αSMA are also localized around annular defects [66] and these may represent an endogenous repair response. Fragmentation of the SLRP proteoglycans biglycan and fibromodulin is further evidence of the tissue remodeling that occurs with disc degradation [86]. Microscopic examination of the annular attachment sites in the CEP to the vertebral body showed a number of structural alterations in response to a controlled outer annular surgical lesion [32] (Figures 1, 2, 3). A significant increase in the size of the annular lesion to 6 × 20 mm in the ovine model accelerated the induction of IVDD [57]. Administration of mesenchymal stromal stem cells successfully repaired such large lesions, and a return in the proteoglycan content and biomechanical properties of the repaired IVDs occurred, a very significant finding when the large size of the 6 × 20 mm lesion is considered in the ovine IVD model. The reparative properties of mesenchymal stem cells are thus particularly impressive and demonstrate the therapeutic value of stem cells in IVD repair [58, 59].

**TABLE 2:** A historical perspective of the broad impact of the ovine spinal model in pathophysiological studies on the IVD and spinal components in health and disease.

Study	Scope of the study	References
A. The ovine annular lesion model of experimental disc degeneration (IVDD) reproduces degenerative Pathology described in human IVDD. This model is also suitable for the evaluation of the efficacy of mesenchymal stem cells to effect repair and functional recovery of degenerate ovine IVDs		
Osti et al. (1990)	Volvo Prize 1990: Establishment of the ovine annular lesion model of experimental IVDD	[1]
Moore et al. (1992)	Changes in endplate vascularity after an outer annulus tear	[61]
Melrose et al. (1992)	A longitudinal study of IVD ECM changes in the ovine IVD	[56]
Moore et al. (1996)	Remodeling of vertebral bone after outer annular injury in sheep	[62]
Melrose et al. (1997)	Topographical variation in the catabolism of aggrecan in an ovine annular lesion model of experimental IVDD	[85]
Moore et al. (1999)	Osteoarthrosis of the facet joints resulting from annular rim lesions in sheep lumbar discs	[61]
Fazzalari et al. (2001)	Mechanical, pathologic consequences of induced concentric annular tears in the ovine model	[104]
Martini et al. (2001)	Use of ovine model in orthopedic research	[105]
Melrose et al. (2002)	Increased nerve and blood vessel ingrowth following proteoglycan depletion in an ovine annular lesion model of experimental disc degeneration	[65]
Melrose et al. (2002)	Spatial and temporal localization of TGF‐β, FGF‐2, and α‐SMA in the injured AF: Implications for ECM repair	[66]
Fagan et al. (2003)	ISSLS Prize 2003 Quantitative analysis of IVD innervation	[106]
Shen et al. (2003)	Grammer Prize 2003 Induction of MMP‐2 and 3 in ovine NP by IL‐1 beta: a potential pathway of disc degeneration	[107]
Thompson et al. (2004)	The mechanical effects of ovine IVD lesions	[108]
Melrose et al. (2007)	Biglycan and fibromodulin fragmentation, temporal and spatial annular remodeling in experimentally injured ovine IVDs	[86]
Schollum et al. (2010)	Examination of alterations in normal annular structure induced by mechanical destabilization in the ovine model	[32]
Melrose et al. (2012)	Mechanical destabilization induced by controlled annular incision of the IVD dysregulates MMP expression and induces IVDD	[57]
Shu et al. (2017)	Quantitative histopathological scoring of IVDD and the utility of adult mesenchymal stem Cells for IVD repair	[58]
Shu et al. (2018)	Efficacy of administered mesenchymal stem cells in the initiation and co‐ordination of IVD repair processes in ovine IVDD	[59]
Wang et al. (2018)	A novel large animal model of lumbar spinal joint degeneration	[109]
Long et al. (2018)	Effects of level, loading rate, injury and repair on biomechanical response of ovine cervical IVDs	[110]
Long et al. (2019)	Morphological/biomechanical effects of AF injury and repair	[111]
Bouhsina et al. (2021)	Correlation between magnetic resonance, X‐ray imaging alterations and histological changes in an ovine model of age‐related disc degeneration	[112]
Decante et al. (2021)	Collateral effects of targeting the nucleus pulposus via a transpedicular or transannular surgical route: a combined X‐ray, MRI, and histological long‐term descriptive study in sheep	[113]
Deneuville et al. (2021)	Quantitative MRI to characterize the nucleus pulposus morphological and biomechanical variation according to sagittal bending load and radial fissure, an ex vivo ovine specimen proof‐of‐concept study	[114]
Jones et al. (2021)	Open‐source image analysis software yields reproducible MRI measures of lumbar intervertebral disc degeneration in sheep models	[115]
Friedmann et al. (2021)	Intervertebral disc regeneration injection of a cell‐loaded collagen hydrogel in a sheep model	[116]
Borem et al. (2021)	Characterization of chondroitinase‐induced lumbar intervertebral disc degeneration in a sheep model intended for assessing biomaterials	[117]
Saghari et al. (2022)	A biodegradable polymeric matrix for the repair of annulus fibrosus defects in intervertebral discs	[118]
Bouhsina et al. (2022)	Comparison of MRI T1, T2, and T2* mapping with histology for assessment of intervertebral disc degeneration in an ovine model	[119]
Page et al. (2022)	Biomechanical evaluation of a novel repair strategy for intervertebral disc herniation in an ovine lumbar spine model	[120]
Li et al. (2022)	Evaluation of the efficacy of stem cell therapy in animal models of intervertebral disc degeneration based on imaging indicators	[121]
Constant et al. (2022)	Comparison and optimization of sheep in vivo intervertebral disc injury model	[122]
Farrugia et al. (2020)	Spatiotemporal expression of 3‐B‐3(−) and 7‐D‐4 chondroitin sulfation, tissue remodeling, and attempted repair in an ovine model of intervertebral disc degeneration	[123]
Poletto et al. (2023)	Preclinical in vivo animal models of intervertebral disc degeneration	[124]
Alini et al. (2023)	An update on animal models of IVDD and LBP	[53]
Gkantsinikoudis et al. (2024)	Morphometric, biomechanical, and histologic assessment of physiologic ovine cervical intervertebral disc	[125]
Melrose and Guilak (2024)	Diverse and multifunctional roles for perlecan (*HSPG2*) in repair of the intervertebral disc	[126]
B. Neurophysiological and biomechanical responses of paraspinal muscles to spinal manipulation: altered responses in IVDD, and cellular changes in paraspinal muscles affecting spinal flexibility		
Colloca et al. (2000)	Neurophysiologic response to lumbosacral spinal manipulation	[127]
Keller et al. (2003)	Neuromechanical characterization of in vivo lumbar spinal manipulation. Part I. Vertebral motion	[128]
Colloca et al. (2003)	Neuromechanical characterization of in vivo lumbar spinal manipulation. Part II. Neurophysiological response	[129]
Colloca et al. (2006)	Spinal manipulation force/duration affect vertebral movement	[130]
Clarke et al. (2007)	Immature sheep spines are more flexible than mature spines	[131]
Colloca et al. (2007)	IVDD reduces vertebral motion responses in an ovine model	[132]
Colloca et al. (2008)	IVDD effects on neurophysiological responses in lumbar spine	[133]
Colloca et al. (2009)	Validation of noninvasive dynamic spinal stiffness methodology	[134]
Colloca et al. (2012)	Biomechanical quantification of pathologic manipulable spinal lesions: an in vivo ovine model of spondylolysis and IVDD	[134]
Hodges et al. (2014)	Proinflammatory cytokine induced multifidus muscle fiber changes after an intervertebral disc lesion?	[77]
Hodges et al. (2015)	Multifidus muscle structural remodeling in IVDD spines	[78]
James et al. (2016)	MSC treatment of IVDD prevents fatty infiltration and fibrosis of the multifidus but not cytokine and muscle fiber changes	[79]
James et al. (2018)	Macrophage polarization contributes to local inflammation and structural change in the multifidus muscle after IVD injury	[80]
James et al. (2022)	Multifidus muscle spindle structural change after IVDD	[81]
James et al. (2024)	Targeted multifidus muscle activation reduces fibrosis of multifidus muscle following IVDD	[92]
James et al. (2025)	Structural changes in muscle spindles of the multifidus muscle after IVDD are resolved by targeted activation of the muscle	[93]
C. Studies on the micro‐structure of the ovine IVD, interlamellar cohesivity, dynamic visco‐elastic stabilization of the composite disc structure and anchorage of the IVD to vertebral endplates		
Pezowicz et al. (2005)	Intralamellar relationships within the collagenous AF	[135]
Pezowicz et al. (2006)	Structural basis of interlamellar cohesion in the IVD	[136]
Schollum et al. (2008)	ISSLS prize 2008 Microstructural and mechanical disruption of the lumbar AF translamellar bridging networks	[88]
Schollum et al. (2009)	A microstructural investigation of IVD lamellar connectivity	[89]
Veres et al. (2008)	ISSLS prize 2008. Microstructural and mechanical disruption of the lumbar disc AF under hydrostatic pressure	[137]
Veres et al. (2010)	ISSLS prize 2010. Loading rate influences disc failure mechanics	[138]
Veres et al. (2010)	Influence of torsion on disc herniation combined with flexion	[139]
Wade et al. (2011)	A fresh look at the nucleus‐endplate structural integration	[140]
Wade et al. (2012)	On the extent and nature of nucleus‐annulus integration	[141]
Wade et al. (2012)	NP‐endplate integration at the fibrillar level in the ovine IVD	[142]
Rodrigues et al. (2012)	Micromechanics of AF‐CEP integration in the IVD	[90]
Wade et al. (2014)	Biomechanical and microstructural study of combined effects of compression rate and flexion on IVD herniation	[143]
Wade et al. (2015)	“Surprise” flexion loading increases the risk of IVD herniation due to AF‐CEP junctional failure	[144]
Rodrigues et al. (2015)	Multiscale structural investigation of AF‐CEP anchorage system	[67]
Wade et al. (2016)	ISSLS Prize 2017 vibration really does disrupt the disc	[145]
Wade et al. (2017)	IVD herniation model incorporating compression, flexion and facet‐constrained shear: Part I: Low rate loading	[71]
Shan et al. (2017)	IVD herniation model incorporating compression, flexion and facet‐constrained shear Part II: high rate or “surprise” loading	[70]
Rodrigues et al. (2017)	How maturity influences annulus‐endplate integration in the ovine intervertebral disc: a micro‐ and ultra‐structural study	[91]
Schollum et al. (2018)	Influence of concordant complex posture and loading rate on motion segment mechanical and microstructural failure	[146]
Schollum et al. (2018)	Microstructural investigation of IVDD induced by low frequency cyclic loading	[147]
Zhang et al. (2019)	Ratcheting behavior of IVDs under cyclic compression	[148]
Wade et al. (2020)	Disc wall structural abnormality initiation sites for herniation	[149]
Wade et al. (2022)	AF defects can act as initiation sites for herniation	[150]
Yang et al. (2019)	Creep‐vibration‐compression study on the lumbar IVD	[151]
Li et al. (2020)	Strain rate mechanical failure of lumbar IVD under flexion	[152]
Liu et al. (2024)	Mechanical properties of lumbar IVD under high loading rate	[153]
Page et al. (2022)	Novel repair strategy for IVD herniation in an ovine IVD model	[120]
Öztürk et al. (2024)	Impact of complex loadings on L2‐L3 IVD structure	[154]
Menghoni et al. (2015)	Inter‐lamellar behavior of the intervertebral disc annulus	[155]
Menghoni et al. (2015)	Modeling failure mechanism of lamellar AF tissues	[156]
Yu et al. (2016)	Role of PLL in cervical disc replacement	[157]
Stewart et al. (2017)	Age‐related differences in intralamellar biomechanics	[158]
McLain et al. (2002)	Comparative morphometry of L4 vertebrae	[159]
D. Ovine studies on structure–function inter‐relationships of elastic fibers and their roles in composite IVD viscoelastic behavior		
Tavaloki et al. (2017)	Ultrastructural organization of IVD elastic networks	[160]
Tavaloki and Costi (2018)	Ultrastructural organization of elastic fibers in the AF of the IVD	[161]
Tavaloki et al. (2018)	Biomechanics of the inter‐lamellar matrix during lumbar disc herniation: which is the weakest structure?	[162]
Tavaloki and Costi (2018)	Viscoelastic behavior of inter‐lamellar AF in radial and circumferential directions upon loading	[163]
Tavaloki and Costi (2018)	Viscoelastic and failure mechanics of elastic fiber AF networks	[164]
Tavaloki and Costi (2018)	Visualization and isolation of elastic fibers in AF of the disc	[165]
E. Studies on the impact of annular lesions and spinal curvature on the functional properties of the ovine IVD		
Ahlgren et al. (1994)	An assessment of the annular incision technique on the strength and multidirectional flexibility of the healing ovine IVD	[166]
Oda et al. (1999)	Spinal kyphotic deformity effects on spinal motion segment	[167]
Ahlgren et al. (2000)	Effect of annular repair on the healing strength of the ovine IVD	[168]
Fu et al. (2016)	Effect of a new annular incision on IVD biomechanics	[169]
Wang et al. (2020)	Lordotic curvature effects adjacent IVDs following spinal fusion	[170]
F. The impact of IVD hydration on intradiscal pressure and the function of the ovine IVD		
Kandziora et al. (2001)	Comparison between sheep and human cervical spines: an anatomic, radiographic, bone mineral density, and biomechanical study	[98]
Costi et al. (2002)	The effect of hydration on the stiffness of ovine IVDs	[171]
Reitmaier et al. (2013)	Preliminary investigations on intradiscal pressures during daily activities: an in vivo study using the merino sheep	[172]
Pei et al. (2014)	Creep bulging deformation of intervertebral disc under axial compression	[173]
Reutlinger et al. (2014)	Specimen specific parameter identification of ovine lumbar IVDs: On the influence of fiber–matrix and fiber–fiber shear interactions	[174]
Daentzer et al. (2015)	In vitro kinematic, intradiscal pressure and biomechanical analyses in cervical arthroplasty versus fusion in a sheep model with two semi‐constrained prostheses	[175]
Tourell et al. (2017)	Load‐induced changes in the diffusion tensor of ovine AF: A pilot MRI study	[176]
Casaroli et al. (2017)	Numerical prediction of the mechanical failure of the IVD under complex loading	[177]
Derrouiche et al. (2019)	Osmo‐inelastic response of the IVD	[178]
G. Use of the Ovine spinal model in a range of spinal fusion studies and in fracture repair to evaluate biological approaches, interbody cages and end‐plate fixation in spinal fusion studies and to evaluate metastatic collapse of spinal tissue		
Magin and Delling (2001)	Improved lumbar vertebral interbody fusion using rhOP‐1: a comparison of autogenous bone graft, bovine hydroxylapatite (Bio‐Oss), and BMP‐7 (rhOP‐1) in sheep	[179]
Kandziora et al. (2002)	Influence of cage design on interbody fusion in a sheep cervical spine model	[180]
Kandziora et al. (2002)	Use of BMP‐2 in a poly(D,L‐lactide)‐coated interbody cage: in spinal fusion	[181]
Kandziora et al. (2002)	IGF‐I and TGF‐beta1 use in a poly‐(D,L‐lactide)‐coated cage promotes vertebral bone matrix formation in an ovine spinal fusion model	[182]
Kandziora et al. (2002)	Comparison of BMP‐2 and combined IGF‐I/TGF‐β in a sheep cervical spine fusion model	[183]
Kandziora et al. (2003)	Dose‐dependent effects of combined IGF‐I/TGF‐β in an ovine cervical spinal fusion model	[184]
Ebihara et al. (2004)	A biomechanical analysis of metastatic vertebral collapse of the thoracic spine in sheep	[185]
Kandziora et al. (2004)	Bioabsorbable interbody cage development in a sheep cervical spine fusion model	[186]
Lee et al. (2006)	Biomechanical evaluation of spinal fixation/healing in an ovine destabilized cervical spine model: a comparison of anterior plating and posterior wiring techniques	[187]
Vresilovic et al. (2006)	Disc mechanics with trans‐endplate partial nucleotomy are not fully restored following cyclic compressive loading and unloaded recovery	[188]
Heineck et al. (2010)	The lumbar sheep spine as a fracture model	[189]
Lyons et al. (2011)	Failure of resorbable plates and screws in an ovine model of anterior cervical discectomy and fusion	[190]
Stieber et al. (2011)	The facet joint loading profile of a cervical IVD replacement incorporating a novel saddle‐shaped articulation	[191]
Sinclair et al. (2013)	The significance of calcified fibrocartilage on the cortical endplate of the translational sheep spine model	[192]
Nuss et al. (2006)	Biocompatability testing of biomaterials	[193]
H. A comparison of ovine IVDs with other animal IVDs and the human IVD using a number of methods and imaging modalities to demonstrate the applicability of the ovine IVD for valid comparisons with the human IVD		
Schmidt & Reitmaier (2013)	Is the ovine IVD a small human one? A finite element model study	[194]
Kettler et al. (2007)	Evaluation of calf, pig, and sheep spinal models for pre‐clinical implant tests?	[195]
Beckstein et al. (2008)	Animal and human IVDs for Spinal research, comparison of axial compression mechanics and glycosaminoglycan content	[196]
Easeley et al. (2008)	Biomechanical and radiographic evaluation of an ovine model for comparisons with the human lumbar spine	[197]
Showalter et al. (2012)	Comparison of experimental animal and human IVDs: evaluation of torsion mechanics and collagen content	[198]
Mageed et al. (2013)	Morphometrical comparison of the ovine and human IVD	[199]
Monaco et al. (2016)	A comparison between porcine, ovine, and bovine IVD anatomy and single lamella AF tensile properties	[200]
Casaroli et al. (2017)	A novel finite element model of the ovine lumbar IVD with anisotropic hyperelastic material properties	[101]
Casaroli et al. (2018)	Finite element comparison between the human and the ovine lumbar IVD	[201]
Sharabi et al. (2018)	3D microstructural reconstruction of the ovine IVD using ultrahigh field MRI	[69]
Bashkuev et al. (2019)	Is the sheep a suitable model to study the mechanical alterations of disc degeneration in humans? A probabilistic finite element model study	[100]
Deneuville et al. (2021)	Quantitative MRI to characterize the nucleus pulposus morphological and biomechanical variation according to sagittal bending load and radial fissure, an ex vivo ovine specimen proof‐of‐concept study	[114]

### 5.1 Fine IVD Structure and How It Provides an Explanation for IVD Functional Properties

A comprehensive series of studies has examined the fine microscopic organization of normal ovine IVD tissues [32, 67, 70, 88, 89, 91, 135, 136, 137, 138, 141, 142, 146, 147, 148, 149, 150, 151], demonstrating how the cohesivity of IVD tissues is achieved and how collagenous and elastic fibers stabilize IVD tissues and annular attachment to the vertebral bodies [160, 161, 162, 163, 164, 165] important in the coordinated functional properties of spinal functional units. This has also allowed the contribution of facet joints to spinal stability to be evaluated [61, 70, 71, 144]. Disruption in IVD structural components by abnormal loading or vibration has also been evaluated in the ovine spine [145, 151]. The importance of the hydration of spinal tissues on intradiscal pressure and how this affects IVD functional properties has also been evaluated in the ovine spine [171]. The ovine IVD model has been used to evaluate different loading regimens on the initiation of IVD degeneration [70, 71, 146, 154]. Two ovine studies have emphasized the importance of compression, flexion, and facet‐constrained shear forces in novel models of IVD degeneration [70, 71]. Thus, the interaction of the CEP, IVD, and facet joints all has potential roles in the mechanism of disc degeneration. This fits in with the hypothesis that rim lesions had initiating roles in disc degeneration and was the reason why the annular lesion model was developed 35 years ago to test this hypothesis.

### 5.2 Spinal Fusion Studies on the Ovine Spine

The ovine spine has proven useful for evaluation of spinal fusion procedures and biological approaches and implant cages to improve fusion processes. The ovine spine has also been used as a model of fracture repair and has been used extensively for teaching purposes in orthopedic procedures [179, 183, 184, 185, 186, 187, 188, 189, 190, 191, 192, 193].

### 5.3 Comparison of Animal IVD Structure Using MRI

A number of studies have compared the ovine IVD with other animal IVDs used in IVD studies and with the human IVD and generally concluded the lumbar ovine IVD shows close comparison with the human IVD [195, 196, 197, 198, 199, 200]. Finite element models of the ovine IVD have been developed for comparisons with the human IVD [101, 201]. Ultra‐high field MRI has been used in microstructural reconstructions of the ovine IVD and in the development of quantitative MRI methods for the analysis of IVD behavior during axial loading and sagittal bending and in IVDs affected by radial fissures [69, 101, 114, 201].

## 6 Future Research

### 6.1 Promising New Therapeutic Approaches to the Treatment of IVDD

As already discussed, the development of new effective treatments for the alleviation of IVDD and LBP is an important research imperative, LBP is the number one global musculoskeletal condition with high socioeconomic impact. Animal models of IVDD have made important contributions aiding in our understanding of IVD degenerative processes.

### 6.2 Efficacy of Mesenchymal Stem Cells for the Repair of IVD and Paradiscal Tissues

**FIGURE 5:**
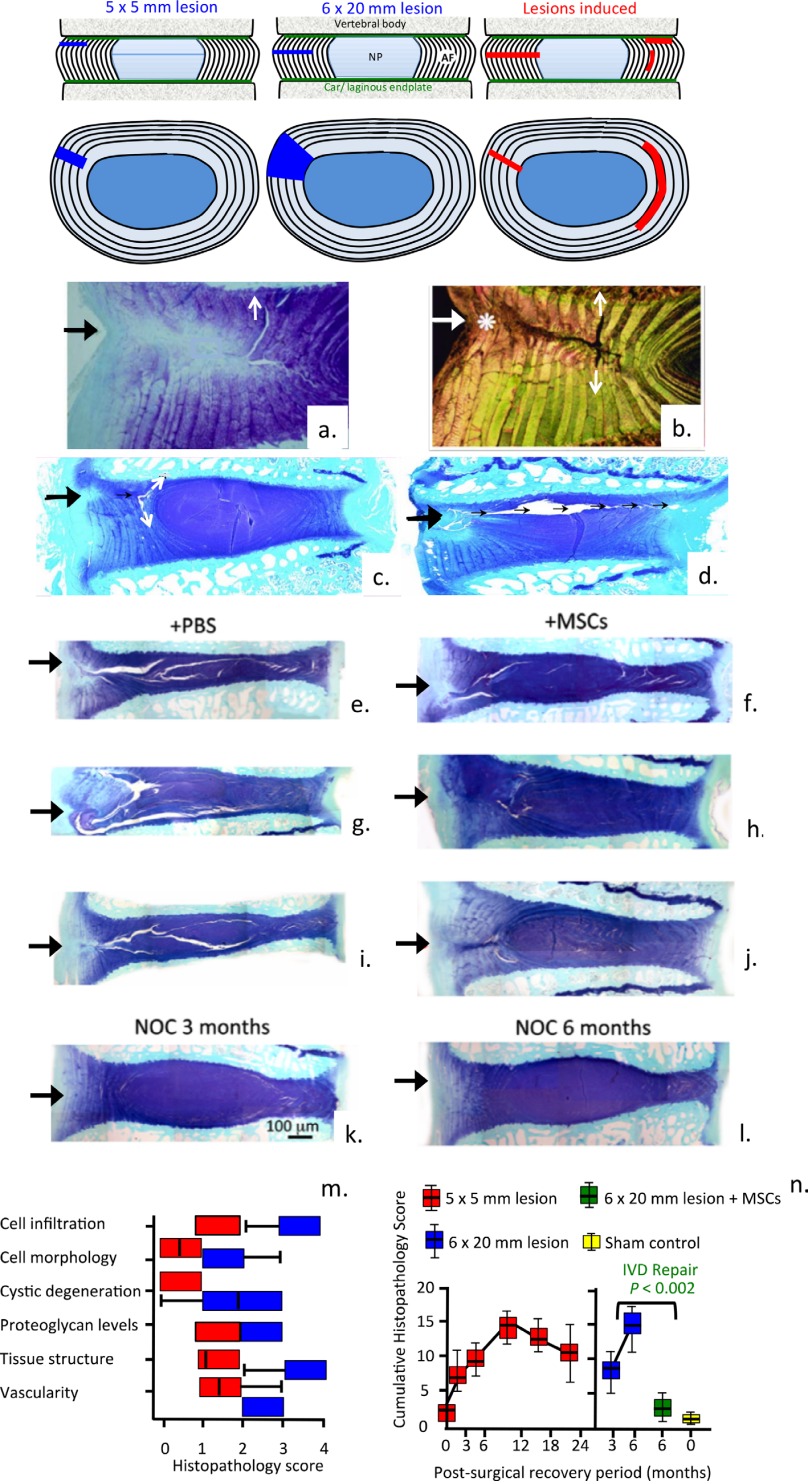
Annular tears are prominent features of degenerate IVDs, MSCs promote repair of these defects. Composite figure depicting schematic representations of the 5 × 5 mm and 6 × 20 mm ovine annular lesions and defects which occur during development of IVDD. Histology of 5 × 5 mm lesion affected IVDs stained with toluidine blue‐fast green demonstrating proteoglycan depletion in the lesion site (a, c, d) and picrosirius staining to examine collagen organization viewed under polarized light (b), regions of annular de‐lammelation (white arrows) are also shown and the propagation of a lesion through to the contralateral AF (black arrows), and inversion of normal annular lamellar structure in the outer AF (*). Toluidine blue stained IVDs which received 6 × 20 mm annular lesions and IVDD was induced for 3 months (e–j) and non‐operated control (NOC) IVDs 3 and 6 months after induction of the lesion (k, l). A number of lesion IVDs were administered mesenchymal stem cells at 3 months and IVD repair was allowed to proceed for a further 3 months (f, h, j). The original lesion site is depicted with a black arrow at the LHS of figures. Histopathological scoring of 5 × 5 and 6 × 20 mm lesions and MSC administered IVDs (+MSCs) (m, n) generation of degenerative pathology and reversal by MSCs. Image a, b reproduced from [87]. Images c–m reproduced from [59] and [58] with permission.

Mesenchymal stem cells show considerable promise for the treatment of IVDD and LBP [59, 202, 203, 204, 205, 206, 207, 208] (Figure 5). Intradiscal administration of mesenchymal stem cells (MSCs) into degenerate ovine IVDs has been highly successful in IVD repair, re‐attainment of IVD disc height and recovery of IVD mechanical properties [58, 59].

### 6.3 IVDD and How It Impacts Spinal Muscle Systems and Spinal Flexibility

Examination of multifidis muscle from MSC treated IVDs showed that this prevented increased deposition of adipose and connective tissue in the multifidis but did not prevent slow‐to‐fast muscle fiber type transformation. Elevated IL1β gene expression in the multifidis of MSC treated IVDs was suppressed however TNFα and TGFβ1 were upregulated after 6 month treatment with MSCs [79]. A sheep model of IVD degeneration has also been used to investigate the role of macrophages and TNFα in the structural alterations that occur in the multifidus muscle when the IVD undergoes degenerative changes [80]. Inflammatory processes have been proposed to regulate subacute/early chronic phases of tissue modeling with the balance between pro‐inflammatory (M1) and anti‐inflammatory (M2) macrophage populations contributing to the maintenance of tissue integrity following injury. TNFα expression by M1 macrophages is elevated in adipose and connective tissue in the multifidis of spines containing degenerate IVDs, these have active roles in subacute/early chronic remodeling in muscle, adipose and connective tissues of the multifidus during IVD degeneration [80]. Further examination of the muscle fiber organization in the remodeling multifidis in spines containing degenerate IVDs has identified alterations in the muscle spindles located centrally and an increase in connective tissue surrounding the spindles containing collagen I and III [81]. This change in capsule stiffness may impact on the transmission of sensory information and may explain some of the proprioceptive deficits identified with LBP. Muscle spindles are stretch detectors, and when activated cause the muscle being stretched to generate tension to resist the stretch alterations in the muscle spindles.

### 6.4 Bioactive Plant Compounds and Related Molecules as Prospective Therapeutic Agents in IVDD

#### 6.4.1 Plant Compounds

A large number of bioactive plant compounds have shown potential in the treatment of IVDD [209, 210]. Plants have been used for healing purposes in traditional Chinese medicine for at least 3000 years [211] and their antioxidant, anticarcinogenic, antiallergenic, anti‐inflammatory, antimutagenic, and antimicrobial activities have been harnessed in biomedicine [212]. Degeneration of the IVD is a multifactorial disease of considerable complexity involving at least 10 cell signaling pathways. Plant compounds can target specific aspects of these cell signaling pathways (Table 3).

**TABLE 3:** Therapeutic properties of natural plant compounds of potential application in the treatment of IVDD.

Compound	Major cell signaling pathways effected	Therapeutic effect
Flavonoids	NF‐kB	Inflammation ↓
Terpenoids	NF‐kB PI3K/Akt	Inflammation ↓ ECM stabilization ↑
Glycosides	SIRT/Nrf2	Apoptosis ↓
Phenolics	SIRT/Nrf2	Apoptosis ↓
Alkaloids	MAPK (p38/JNK/ERK) AMPK/mTOR	Cell viability ↑ Autophagy ↑

Abbreviations: Akt, protein kinase B; AMPK, Adenosine monophosphate activated protein kinase; ERK, extracellular signal‐regulated kinase; JNK, c‐Jun N‐terminal kinase; MAPK, mitogen‐activated protein kinase; mTOR, mammalian target of rapamycin; NF‐kB, nuclear factor kappa‐light‐chain‐enhancer of activated B cells; Nrf2, nuclear factor erythroid 2‐related factor 2; p38, mitogen‐activated protein kinase; PI3K, phosphatidylinositol 3‐kinase; SIRT, Sirtuin 1, NAD‐dependent deacetylase.

Over 10 000 flavone and flavonoid compounds have been characterized [213] of potential therapeutic value in IVDD [209, 214, 215, 216, 217, 218], 40 000 Terpenoid structures have also been described, and phenolic, alkaloid and glycoside compounds display a considerable range of therapeutic cell regulatory properties in inflammation, apoptosis, ECM stabilization, cell viability and autophagy of relevance to the treatment of IVDD. It was beyond the scope of this study to cover all these compounds however the interested reader is referred to a number of excellent reviews on these compounds and their uses in the treatment of IVDD [209, 214, 215, 216, 217, 218].

#### 6.4.2 The Statins and Animal Models of Experimental IVDD

The statins are fungal cholesterol lowering drugs [219, 220] that are also beneficial in the treatment of IVDD, inhibiting degenerative changes in the IVD and stimulating repair [221, 222, 223]. Lovastatin upregulates BMP‐2 and SOX9 expression and promotes chondrogenesis in rat caudal discs [223]. Simvastin promotes IVD repair processes in a rat model of IVDD [224] upregulating BMP2 expression and stimulates chondrogenic processes in experimental IVDD [224]. Rosuvastatin inhibits mechanical pressure‐induced IVDD [225]. Rats fed a high cholesterol diet display degenerative features in lumbar IVDs that can be abolished by atorvastatin.

#### 6.4.3 Pro‐Resolving Anti‐Inflammatory Lipids Rescue the Functional Properties in Degenerate IVDs

Specialized pro‐resolving mediators (SPMs) promote the resolution of inflammation promoting disc health [226, 227]. Lipoxin (LXA4) and anti‐inflammatory resolvins, protectins, and maresins inhibit the production and action of IL‐6, TNF‐α, and other pro‐inflammatory cytokines in IVDD [227, 228]. This prevents excessive inflammation and restores tissue homeostasis. Resolvin D2 suppresses the expression of IL‐1β and its secretion by macrophages and deactivates the NLRP3 inflammasome. Injections of LXA4 and other SPMs have been proposed for the treatment of IVDD. Peroxidation of lipids generates ROS and inflammatory conditions in IVDD; LXA4, resolvins, and maresins inhibit this [229].

Examination of the therapeutic properties of the compounds discussed above as potential agents for the treatment of IVDD has so far only been conducted in rodent models of IVDD. It is essential that these should be evaluated in a disc model more similar in structure to the human IVD and which contains similar IVD resident cell populations to those found in the human IVD. This would provide data more applicable to the treatment of human IVDD. The ovine model represents such a model; such studies are eagerly anticipated and are predicted to provide valuable supportive information on the use of these promising compounds for the treatment of IVDD in humans.

## 7 Conclusions

The ovine IVD model has made an important contribution to furthering our understanding of the complexity of the multifactorial events that contribute to IVDD and the generation of LBP, the number one musculoskeletal condition. This complexity indicates that a paradigm shift in experimental approaches may be required to fully understand the disc degenerative process in order to develop new methodologies to better select the most appropriate therapeutic targets to effect IVD repair and regeneration and alleviate LBP. The use of mesenchymal stem cells [58, 59, 208] and senolytic drugs [216, 218, 230, 231, 232] represents two new interesting experimental approaches which show considerable promise in the treatment of IVDD. Artificial intelligence has significantly improved spinal imaging evaluations and may also be applicable to the systematic analysis of histochemical and other imaging modalities on IVD tissues [53]. There is also now considerable interest in biological agents for disc repair, and there are several candidates that deserve further evaluation [216, 218, 230, 231, 232]. The ovine model is suitable for such evaluations and may be instrumental in the development of novel IVD repair strategies in the future.

## Author Contributions

J.M. conceived the study, wrote initial drafts, and was assisted by C.B.L. and O.O. in subsequent revisions of the text. All authors approved the final version of the manuscript.

## Conflicts of Interest

This study was funded by The Melrose Personal Research Fund, Sydney, Australia. The authors have no conflicts or disclosures to make.

## Data Availability

The data that support the findings of this study are available on request from the corresponding author. The data are not publicly available due to privacy or ethical restrictions.
